# Preserved Proactive Control in Ageing: A Stroop Study With Emotional Faces vs. Words

**DOI:** 10.3389/fpsyg.2019.01906

**Published:** 2019-09-03

**Authors:** Natalie Berger, Anne Richards, Eddy J. Davelaar

**Affiliations:** Department of Psychological Sciences, Birkbeck, University of London, London, United Kingdom

**Keywords:** proactive control, cognitive control, ageing, task-relevant emotion, Stroop task

## Abstract

Previous studies regarding age-related changes in proactive control were inconclusive and the effects of emotion on proactive control in ageing are yet to be determined. Here, we assessed the role of task-relevant emotion on proactive control in younger and older adults. Proactive control was manipulated by varying the proportion of conflict trials in an emotional Stroop task. In Experiment 1, emotional target faces with congruent, incongruent or non-word distractor labels were used to assess proactive control in younger and older adults. To investigate whether the effects of emotion are consistent across different stimulus types, emotional target words with congruent, incongruent or obscured distractor faces were used in Experiment 2. Data from this study showed that older adults successfully deployed proactive control when needed and that task-relevant emotion affected cognitive control similarly in both age groups. It was also found that the effects of emotion on cognitive performance were qualitatively different for faces and words, with facilitating effects being observed for happy faces and for negative words. Overall, these results suggest that the effects of emotion and age on proactive control depend on the task at hand and the chosen stimulus set.

## Introduction

Research suggests that the ability to exert cognitive control over incoming information is not a unitary process. According to the dual mechanisms of control (DMC) theory (Braver et al., 2007, 2009), there are at least two separable factors: proactive control refers to sustained control, which is recruited before the occurrence of conflict (Braver, 2012), whereas reactive control refers to transient control processes that are recruited once conflict has been detected (Botvinick et al., 2001). In recent years, research started to assess the effects of emotion on these two control modes (Kalanthroff et al., 2016; Grimshaw et al., 2017; Kar et al., 2017). However, none of these studies have investigated the effects of emotion on proactive control in ageing despite evidence of age-related changes in executive functions and in emotional functioning. The aim of the present research was therefore to investigate younger and older adults’ ability to exert proactive control in two emotional Stroop tasks.

### Age-Related Changes in Cognitive and Emotional Functioning

Research indicates that reactive control is preserved in aging (Paxton et al., 2006; Braver, 2012). In contrast, research findings regarding age-related differences in proactive control have been mixed. Significantly impaired goal maintenance was found in older relative to younger adults (e.g., Braver et al., 2005, 2009; Haarmann et al., 2005; Paxton et al., 2008, Exp. 1), which was interpreted as evidence for impaired proactive control in ageing. Other studies, however, reported intact (e.g., Paxton et al., 2008, Exp. 2; Staub et al., 2014) or even improved proactive control in older relative to younger adults (Staub et al., 2014). It should be noted that impaired proactive control in ageing was found in studies using the AX-Continuous Performance Task (AX-CPT; Rosvold et al., 1956), which requires participants to maintain goal-related information and to make target responses on cued trials and non-target responses on all other trials. This task not only tackles proactive control but also requires participants to remember a two-fold set of rules and to keep track of preceding items in order to make correct target and non-target responses to an X. Previous research has shown that these abilities are impaired in ageing: Older adults were found to show difficulties in maintaining two different tasks in working memory (Verhaeghen and Cerella, 2002; Reimers and Maylor, 2005; Wasylyshyn et al., 2011) and in working memory updating (Van der Linden et al., 1994; Hartman et al., 2001; Salthouse et al., 2003; De Beni and Palladino, 2004; Chen and Li, 2007; Schmiedek et al., 2009). Thus, age-related differences in AX-CPT performance might be found due to impairments in processes other than proactive control.

So far, the effects of emotion on proactive control in ageing have received little attention, despite evidence that emotion-cognition interactions change with age (for comprehensive reviews, see Mather, 2004; Mather and Carstensen, 2005; Murphy and Isaacowitz, 2008; Kensinger, 2009). Research from the domain of WM has shown that older adults can benefit from the inclusion of emotional and particularly positive material (Mikels et al., 2005; Mammarella et al., 2013a, b). For instance, Mikels et al. (2005) found age-related impairments in a delayed-response task when participants had to compare the brightness of two neutral pictures but not when they had to compare the emotional intensity of two emotional pictures. Moreover, older adults outperformed younger adults when they had to compare the emotional intensity of positive pictures, whereas younger adults showed better performance than older adults on trials with negative pictures. Age-related impairments were also found when neutral but not when emotional words were used in a modified version of the operation WM span test, in which participants had to maintain words while solving mathematical operations (Mammarella et al., 2013a, b).

Age-related changes in emotion-cognition interactions are usually interpreted within the socioemotional selectivity theory (SST; Carstensen, 1993), according to which older adults use cognitive resources to direct their attention to emotional and particularly positive information to enhance their well-being (for reviews, see Scheibe and Carstensen, 2010; Reed and Carstensen, 2012). It was found that cognitive load can eliminate this emotional bias in ageing (Mather and Knight, 2005), suggesting that older adults’ preference for positive material requires controlled, resource-demanding processes. Based on this assumption, which centers around the availability of cognitive resources, specific hypotheses can be suggested regarding the effects of emotion on cognitive control in ageing. As goal representations are maintained continuously under proactive control, this control mode is thought to be resource-consuming (Braver, 2012) and thus, fewer cognitive resources should be available. If older adults indeed use cognitive resources in order to direct their attention to positive information, it can be expected that a positivity effect in ageing should be less pronounced under conditions requiring high proactive control relative to conditions requiring low proactive control.

### Proactive Control in the Stroop Task

The Stroop task (Stroop, 1935) has been widely used to assess cognitive control. In the classic color version task, color words are printed in a congruent or an incongruent ink color (e.g., “red” printed in red vs. green ink) and participants have to name the color of the ink while ignoring the color word. It is assumed that there is a strong tendency to read the word due to life-long experience with reading (Verhaeghen and De Meersman, 1998) and thus, cognitive control is required to selectively attend to and respond to the weak but task-relevant (i.e., the color of the ink) attribute in the presence of a strong but task-irrelevant (i.e., written color word) attribute (Miller and Cohen, 2001). Typically, incongruent trials are associated with slower responses than non-word trials, a pattern that is known as the Stroop effect (Lindsay and Jacoby, 1994).

However, research suggests that in contrast to non-word trials, not only incongruent but also congruent trials elicit task conflict between word reading and color naming due to the presence of both color and word information (Goldfarb and Henik, 2007, 2013; Kalanthroff et al., 2015; for a review, see Kalanthroff et al., 2013, 2018). Previous studies have used expectancy of task conflict to manipulate the recruitment of proactive control (De Pisapia and Braver, 2006; Funes et al., 2010; Krug and Carter, 2012; Kalanthroff et al., 2015). Goldfarb and Henik (2007), for instance, increased the number of non-word trials (see also Tzelgov et al., 1992) and added cues that informed participants on half of the trials whether the next trial would be a Stroop trial or a non-word trial. On the other half of the trials, the cues were uninformative. This was aimed at reducing or relaxing proactive control in participants on un-cued relative to cued trials, as most of the trials only had task-relevant color information. It was found that on non-cued trials, reaction times (RTs) were longer for congruent compared to non-word trials, which was labeled reversed facilitation. Additionally, RTs were longer for non-cued congruent stimuli compared to cued stimuli and incongruent trials were slower than non-word and congruent trials throughout. These results suggest that participants were less efficient in resolving task conflict on both incongruent and congruent trials when proactive control was low.

Neuroimaging studies also found that conditions with a high expectancy (HE) of conflict (i.e., congruent and incongruent) trials in a Stroop task were associated with sustained activity in the dorsolateral prefrontal cortex (DLPFC) that is linked to the deployment of cognitive control (De Pisapia and Braver, 2006; Krug and Carter, 2012). In contrast, conflict trials under conditions with a low expectancy (LE) of conflict trials were associated with event-related activation of a medial and lateral prefrontal cognitive control network, including the anterior cingulate cortex (ACC), which has been linked to conflict monitoring (De Pisapia and Braver, 2006; Krug and Carter, 2012). Behaviorally, two indices for the recruitment of proactive control in a Stroop paradigm can be used: interference, which is the difference between RTs for incongruent and non-word trials, and facilitation, which is the difference between RTs for congruent and non-word trials. High levels of proactive control under conditions of HE of task conflict are thought to be associated with reduced interference and facilitation. In contrast, low levels of proactive control under conditions of LE of task conflict are thought to be associated with increased interference and no or even reversed facilitation (Tzelgov et al., 1992; Goldfarb and Henik, 2007; Kalanthroff et al., 2013, 2015).

### The Present Research

The aim of this research was to assess the effects of age and emotion on proactive control in two emotional Stroop tasks. Expectancy of task conflict was used to manipulate proactive control and emotional faces and words were used to test whether the role of emotion is consistent across different stimulus sets. Experiment 1 assessed older and younger adults’ ability to exert proactive control in an emotional Stroop task with faces. Although the Stroop task has been used to investigate the effects of emotion on proactive control, emotional items were often included as task-irrelevant distractors (e.g., Kalanthroff et al., 2016; Grimshaw et al., 2017). The effects of task-relevant emotional targets, on the other hand, were often not considered, despite evidence that emotion can improve cognitive performance through enhanced target processing (Pessoa, 2009). In a study by Krug and Carter (2012), for instance, participants responded to the emotion of neutral and fearful faces, while these were shown with congruent and incongruent emotion labels (“neutral” or “fearful”). The authors reported higher interference by an irrelevant emotional (i.e., “fearful”) relative to an irrelevant neutral label distractor. An alternative interpretation, which was not explored by the authors, is that interference was actually reduced for emotional targets (fearful face with irrelevant neutral label) rather than increased for emotional distractors (neutral face with irrelevant emotional label). In another study, Kar et al. (2017) used happy vs. sad (Exp. 1) or happy vs. angry target faces (Exp. 2) with congruent and incongruent distractor labels in a Stroop task and found that conflict adaptation, a measure of proactive control, varied as a function of previously presented emotion. However, neutral faces were not included and this absence of a neutral baseline makes it difficult to interpret differential effects of sad vs. happy or angry vs. happy faces.

## Experiment 1

To address the limitations of previous research, three emotions were included in the present facial Stroop task: happy, neutral, and angry target faces. Based on research showing that happy faces are more efficiently detected than other expressions (Kirita and Endo, 1995; Becker et al., 2011; Becker and Srinivasan, 2014), it was predicted that happy targets would be associated with higher accuracy and faster RTs relative to neutral or angry targets. As research (Carstensen, 1993) suggests that older adults focus on positive material more than younger adults and that this focus requires cognitive resources, it was hypothesized that older adults would show particularly improved performance for happy faces relative to younger adults. However, this was expected under LE conditions requiring low levels of proactive control, as more resources would be available to focus on happy faces relative to HE conditions requiring high levels of resource-demanding proactive control.

### Methods

#### Participants

Thirty younger (19–40 years old) and 30 older adults (62–85 years old) participated in the experiment (see Table 1 for participant characteristics). One younger and one older participant were excluded from the analysis due to RTs that were 2.5 *SD* slower than the respective age group’s mean RTs. Younger adults were undergraduate and postgraduate students at Birkbeck, University of London, and received either course credits or £7.50 per hour for their participation. Older adults were recruited from the University of the Third Age in London and were paid at the same rate as younger adults for their participation. Participants were community-dwelling and were pre-screened for psychiatric disorders and a history of neurological disorders. They reported to be in good health and had normal or corrected-to-normal vision. Older participants had a score of 27 or above on the Mini-Mental State Examination (MMSE; Folstein et al., 1975). Older adults had better verbal knowledge as assessed with the NART (Nelson and Willison, 1991) and showed slower processing speed as measured by the Digit Symbol Substitution Test (Wechsler, 1955). No further differences were observed. The ethics board of Birkbeck, University of London, approved the procedure prior to the start of the study and written informed consent was obtained from each participant.

**TABLE 1:** Participant characteristics, Experiment 1.

	**Younger adults**		**Older adults**		**Group difference**	
**Variable**	***M***	***SD***	***M***	***SD***	***t***	***p***
Age	28.14	6.99	71.34	6.75		
Gender (male/female)	8/21		8/21			
Education (years)	15.83	2.49	16.00	3.22	−0.23	0.820
NART Verbal IQ	105.69	6.56	114.46	6.04	−5.25	<0.001
Digit Symbol Test	64.17	11.90	51.69	11.83	4.01	<0.001
BDI II	5.45	5.03	4.64	4.04	0.66	0.509
STAI Trait Anxiety	35.66	9.64	35.32	8.50	0.14	0.890
MMSE			29.10	1.01		

*NART = The National Adult Reading Test, BDI II = Beck Depression Inventory II, STAI = State-Trait Anxiety Inventory, MMSE = Mini-Mental State Examination.*

#### Materials

**FIGURE 1:**
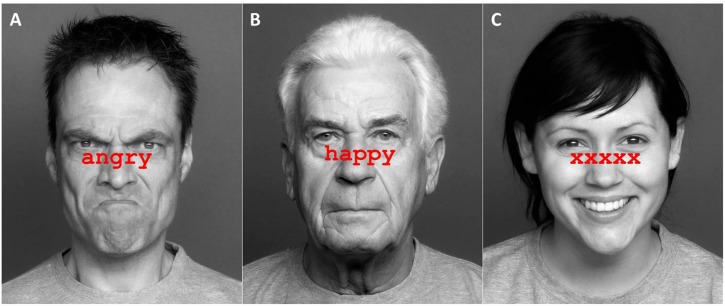
Examples of Stroop stimuli in Experiment 1. Panel **(A)** shows an angry face with a congruent label, panel **(B)** shows a neutral face with an incongruent label, and panel **(C)** shows a happy face with a non-word label. Pictures are taken from the FACES database (Ebner et al., 2010) and can be accessed at: https://faces.mpdl.mpg.de/imeji/. Publication and display of the shown pictures for the purpose of illustrating research methodology are permitted under the FACES Platform Release Agreement.

The stimuli were 36 faces from the FACES database (Ebner et al., 2010), a validated set of photographs of naturalistic faces of different ages in front view. Faces showed angry, neutral or happy expressions (12 items per emotion). The age group (younger, middle-aged, older) and sex (male, female) of the faces were balanced in each emotion category. The faces were taken from a pool of stimuli that had been previously rated by younger and older adults and were selected based on high agreement ratings between both age groups (for evaluation details, see Berger et al., 2017). Congruent items were created by printing matching emotion labels across the emotional faces (e.g., neutral face with “neutral” label). Incongruent items were created by printing non-matching emotion labels across the faces (e.g., angry face with “happy” label). Non-word items were created by printing a string of “xxxxx” across the faces. Combinations of faces and labels are summarized in Table 2. Face images were turned to gray-scale, whilst labels were printed in red, 38-point Courier New font, and placed between eyes and mouths of the faces. Example stimuli are presented in Figure 1.

**TABLE 2:** Combinations of facial expressions and labels that formed congruent, non-word and incongruent stimuli in Experiment 1.

	**Task-relevant facial expression**		
**Distractor label**	**Angry**	**Neutral**	**Happy**
Angry	Congruent	Incongruent	Incongruent
Neutral	Incongruent	Congruent	Incongruent
Happy	Incongruent	Incongruent	Congruent
xxxxx	Non-word	Non-word	Non-word

*Congruent stimuli are color-coded in green, non-word stimuli in yellow and incongruent stimuli in red.*

#### Procedure

After giving informed consent, participants completed a demographic questionnaire and were seated in front of a computer screen. A visual acuity test (Bach, 1996) was conducted at a distance of 65 cm to ensure that vision was in the normal range. Participants were then asked to remain at this distance to the screen and performed the computerized Stroop task, which was prepared and presented using E-Prime Version 2.0.10.353 (Schneider et al., 2002) on a 24-inch computer screen with a resolution of 1920 × 1200 pixels. The task consisted of two blocks, counterbalanced across participants. In the HE block, 75% of the trials were either congruent or incongruent (37.5%, respectively), while 25% of the trials were non-words. In the LE block, 25% of the trials were either congruent or incongruent (12.5%, respectively) and 75% of the trials were non-words. There were equal numbers of angry, neutral, and happy faces across congruent, non-word and incongruent trials as well as across the two blocks. Each block consisted of 288 trials and presentation of trials was random. In each trial, a fixation cross appeared for 500 ms. It was then replaced by the distractor label “angry,” “neutral,” “happy” or “xxxxx,” which was presented for 100 ms. This was done to facilitate label reading, following prior procedures by Krug and Carter (2012). The presentation of the label was followed by the simultaneous presentation of the label and the target face. Participants were instructed to indicate the emotion of the face (angry, neutral or happy) as accurately and quickly as possible by pressing one of three labeled keys. On the computer keyboard, the buttons “1,” “2,” and “3” on the numeric keypad were used. Button presses initiated the presentation of a blank screen for 2000 ms, after which the next trial started. The assignment of emotion labels to buttons was counterbalanced across participants. Participants were instructed to leave the fingers on the buttons for the duration of the task. With the option to take short breaks after every 48 trials, there were five short breaks in each block and one in-between blocks. Participants were tested individually and each session lasted approximately 60–75 minutes in total.

#### Design and Statistical Analysis

Responses and RTs were recorded for each trial and accuracy and median rather than mean RTs for correct trials were calculated for each participant for each condition to account for the skewed distribution of RT data. Statistical analyses of the data were conducted with SPSS 22 (IBM Corp., Armonk, NY). Accuracy and RTs were analyzed by 2 × 3 × 3 × 2 mixed factors ANOVA including the within-subjects factors expectancy (LE vs. HE), congruency (congruent vs. non-word vs. incongruent) and emotion (angry vs. neutral vs. happy) as well as the between-subjects factor of age (younger vs. older). *Post hoc t*-tests with a Bonferroni adjustment to the 5% alpha level were performed to follow up significant main effects and interactions. Due to significant differences in the two age groups’ verbal knowledge and processing speed, all analyses were repeated with NART verbal IQ and Digit Symbol as centered covariates. The results with age as a factor reported here were qualitatively the same and significant in the analysis including covariates. RTs varied considerably between younger and older adults. To guard against spurious interactions between age and experimental conditions due to general slowing in older adults (Faust et al., 1999), log-transformed RTs were used for the analysis (e.g., Kray and Lindenberger, 2000; Tun and Lachman, 2008). To aid interpretation, pre-transformed RTs are reported in the descriptives and figures.

### Results

#### Accuracy

**FIGURE 2:**
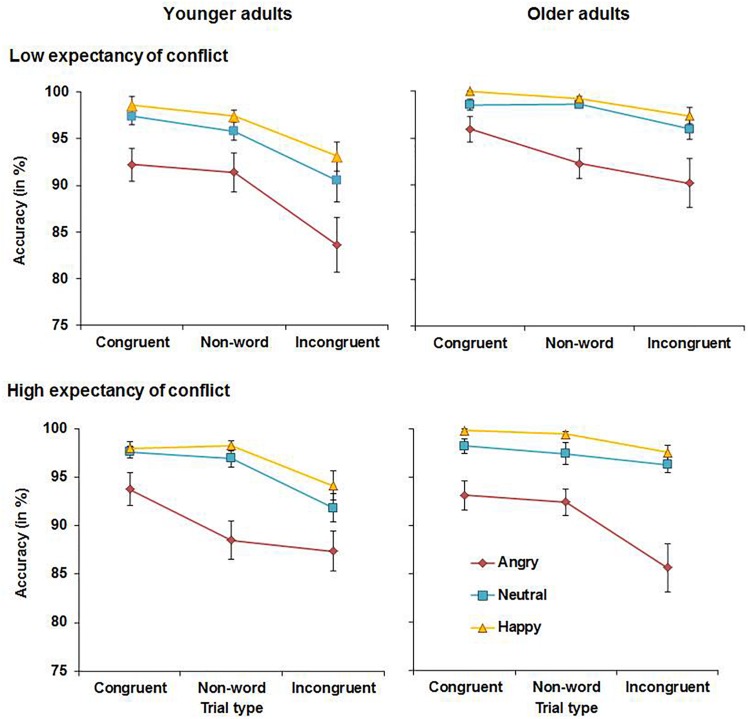
Accuracy in younger adults **(left panels)** and older adults **(right panels)** in Experiment 1.

Accuracy scores for younger and older adults are presented in Figure 2. The analysis yielded a significant main effect of congruency, *F*(2, 112) = 46.23, MSE = 0.007, *p* < 0.001, partial η^2^ = 0.45, with higher accuracy for congruent (*M* = 96.9%, *SD* = 2.7%) compared to non-word (*M* = 95.6%, *SD* = 3.6%), *t*(57) = 3.98, *p* < 0.001, or incongruent trials (*M* = 92.0%, *SD* = 6.4%), *t*(57) = 7.32, *p* < 0.001. Accuracy was also higher for non-word than for incongruent trials, *t*(57) = 6.47, *p* < 0.001. There was also a main effect of emotion, *F*(2, 112) = 29.45, MSE = 0.026, *p* < 0.001, partial η^2^ = 0.34, with higher accuracy for happy faces (*M* = 97.7% *SD* = 3.1%) compared with neutral (*M* = 96.3%, *SD* = 4.2%), *t*(57) = 2.88, *p* = 0.005, or angry faces (*M* = 90.5%, *SD* = 8.5%), *t*(57) = 6.53, *p* < 0.001. Accuracy was also higher for neutral than for angry faces, *t*(57) = 4.81, *p* < 0.001. These main effects were qualified by a significant congruency × emotion interaction, *F*(4, 224) = 4.26, MSE = 0.003, *p* = 0.007, partial η^2^ = 0.07. Follow-up t tests revealed that for angry faces, accuracy was higher for congruent (*M* = 93.8%, *SD* = 7.2%) relative to non-word trials (*M* = 91.1%, *SD* = 8.5%), *t*(57) = 3.80, *p* < 0.001. In contrast, the difference in accuracy between congruent and non-word trials was not significant for neutral (*p* = 0.079) or for happy faces (*p* = 0.102). Accuracy was higher for non-word than for incongruent trials for all three valences (all *t* values ≥ 4.26). There was also a significant expectancy × congruency × emotion × age interaction, *F*(4, 224) = 3.45, MSE = 0.004, *p* = 0.026, partial η^2^ = 0.06. Accuracies under HE and LE conditions were analyzed separately to follow up this interaction. The congruency × emotion × age interaction was non-significant under LE conditions (*p* = 0.560), but was significant under HE conditions, *F*(4, 224) = 5.94, MSE = 0.002, *p* = 0.001, η^2^ = 0.10. Separate analyses for angry, neutral, and happy faces were conducted and while the congruency × age interaction was significant for angry faces, *F*(2, 112) = 3.45, MSE = 0.005, *p* = 0.048, partial η^2^ = 0.06, and for neutral faces, *F*(2, 112) = 5.26, MSE = 0.002, *p* = 0.012, partial η^2^ = 0.07, it was non-significant for happy faces (*p* = 0.237). Follow-up *t*-tests showed different response patterns to angry faces in younger and older adults: Under HE conditions, younger adults showed higher accuracy for congruent (*M* = 93.8%, *SD* = 9.0%) relative to non-word angry faces (*M* = 88.5%, *SD* = 10.5%), *t*(28) = 3.81, *p* = 0.001, and no difference between incongruent (*M* = 87.4%, *SD* = 11.1%) and non-word angry faces (*p* = 0.459). In contrast, older adults showed no difference (*p* = 0.515) in accuracy for congruent (*M* = 93.1%, *SD* = 8.2%) relative to non-word angry faces (*M* = 92.4%, *SD* = 7.5%). Instead, older adults’ accuracy was significantly lower for incongruent (*M* = 85.5%, *SD* = 13.5%) relative to non-word angry faces, *t*(28) = 3.17, *p* = 0.004. Response patterns also differed for neutral faces. In younger adults, accuracy was lower for incongruent (*M* = 91.9%, *SD* = 7.9%) relative to non-word neutral faces (*M* = 97.0%, *SD* = 5.0%), *t*(28) = 3.76, *p* = 0.001, whereas the difference was non-significant in older adults (*p* = 0.239). Lastly, there was also a main effect of age, *F*(1, 56) = 5.77, MSE = 0.024, *p* = 0.020, partial η^2^ = 0.09, driven by higher accuracy in older (*M* = 96.0%, *SD* = 2.9%) than in younger adults (*M* = 93.7%, *SD* = 4.3%). No further significant main effects or interactions were observed for accuracy.

#### Reaction Times

**FIGURE 3:**
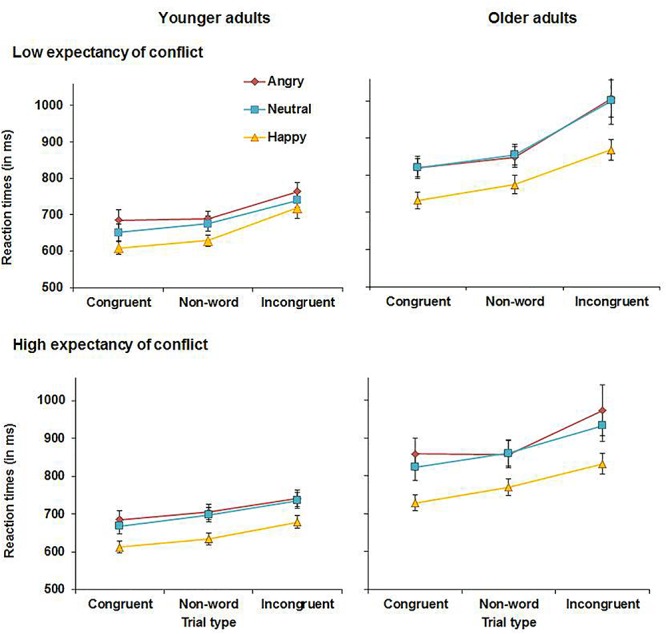
RTs for correct responses in younger adults **(left panels)** and older adults **(right panels)** in Experiment 1.

Reaction times for younger and older adults are presented in Figure 3. The analysis yielded a main effect of congruency, *F*(2, 112) = 124.06, MSE = 0.019, *p* < 0.001, partial η^2^ = 0.69, with overall faster RTs for congruent (*M* = 724 ms, *SD* = 138 ms) than for non-word trials (*M* = 750 ms, *SD* = 137 ms), *t*(57) = 7.89, *p* < 0.001, or incongruent trials (*M* = 833 ms, *SD* = 203 ms), *t*(58) = 12.40, *p* < 0.001. RTs were also faster for non-word than for incongruent trials, *t*(57) = 9.86, *p* < 0.001. This main effect was qualified by an expectancy × congruency interaction, *F*(2, 112) = 12.25, MSE = 0.006, *p* < 0.001, partial η^2^ = 0.18. To follow up on this interaction, the analysis was repeated with the factor congruency only comprising the factor levels congruent and non-word trials and there was no significant expectancy × congruency interaction (*p* = 0.878). In contrast, in the analysis with the factor congruency comprising the factor levels non-word and incongruent trials, there was a significant expectancy × congruency interaction, *F*(1, 57) = 15.87, MSE = 0.006, *p* < 0.001, partial η^2^ = 0.22. Follow-up *t*-tests revealed that under HE conditions, RTs were slower for incongruent (*M* = 815 ms, *SD* = 200 ms) than for non-word trials (*M* = 754 ms, *SD* = 146 ms), *t*(57) = 8.22, *p* < 0.001. Under LE conditions, the difference in RTs between incongruent (*M* = 850 ms, *SD* = 226 ms) and non-word trials (*M* = 745 ms, *SD* = 142 ms) was more pronounced, *t*(57) = 9.95, *p* < 0.001. Moreover, there was a significant main effect of emotion, *F*(2, 112) = 50.61, MSE = 0.022, *p* < 0.001, partial η^2^ = 0.48, and follow-up analyses revealed that RTs for happy faces (*M* = 716 ms, *SD* = 124 ms) were faster than for neutral faces (*M* = 788 ms, *SD* = 177 ms), *t*(57) = 7.36, *p* < 0.001, or angry faces (*M* = 802 ms, *SD* = 181 ms), *t*(57) = 9.20, *p* < 0.001. The difference between RTs for neutral and angry faces was not significant (*p* = 0.139). Lastly, there was also a main effect of age, *F*(1, 56) = 27.32, MSE = 0.421, *p* < 0.001, partial η^2^ = 0.33, as older adults were overall slower (*M* = 853 ms, *SD* = 162 ms) than younger adults (*M* = 684 ms, *SD* = 92 ms). No further significant main effects or interactions were observed for RTs.

### Discussion

Experiment 1 assessed the effects of emotion on proactive control in older and younger adults. Both younger and older adults showed reduced interference in RTs from incongruent relative to non-word trials when expectancy of conflict was high (HE conditions). This suggests that both age groups deployed proactive control under HE conditions, which helped to prime task-relevant processing pathways before the onset of conflict trials. It was also observed that emotional faces affected performance in both age groups. Happy faces improved overall performance as evidenced by higher accuracy and faster RTs for happy compared to neutral or angry faces across conditions with no age-related differences. In contrast, accuracy was lowest and RTs were slowest for angry faces. Younger adults were more accurate when responding to congruent relative to non-word angry faces, whereas older adults showed reduced accuracy for incongruent relative to non-word negative information under HE conditions. Although this could suggest greater impairments in the presence of angry faces in older than in younger adults, this effect was in fact driven by lower accuracy for angry non-word trials in younger than older adults as can be seen in Figure 2. No age-related differences in accuracy were observed for congruent and incongruent angry faces. When presented with neutral faces under HE conditions, younger but not older adults showed lower accuracy for incongruent relative to non-word trials. Thus, there was not only no evidence for age-related impairments in proactive control, but older adults even outperformed younger adults when presented with neutral material under conditions requiring proactive control.

Higher accuracy and faster RTs in the presence of happy relative to neutral or angry faces were observed in both age groups and this is in line with previous research showing improved WM performance for happy faces relative to other expressions (Levens and Gotlib, 2010, 2012; Cromheeke and Mueller, 2015). Enhanced performance for happy faces was found across conditions and did not interact with control in the present research. This indicates that more general processes, for instance emotion recognition, were facilitated by happy faces rather than specific control processes. This is in line with studies showing more accurate and faster recognition of happy relative to other emotional expressions (Juth et al., 2005; Becker et al., 2011; Becker and Srinivasan, 2014). Besides this perceptual advantage it is also likely that happy faces contributed to improved performance due to the rewarding value they carry (O’Doherty et al., 2003; Tsukiura and Cabeza, 2008), which might have facilitated particularly efficient processing of happy faces. In contrast to neutral and angry faces, all happy faces used in this experiment showed teeth, a perceptual cue that could have facilitated recognition of happy faces. Previous research indicates that despite a recognition advantage of open-relative to closed-mouth versions of happy faces, happy expressions are still identified more accurately than other emotional expressions with open or with closed mouth (e.g., Tottenham et al., 2009; Becker et al., 2011).

The facilitating effect of happy faces was not more pronounced in older relative to younger adults, neither in general nor in any of the two conditions, which is not fully in line with the SST (Carstensen, 1993). According to this theory, older adults focus on positive information in order to improve wellbeing, which is reflected in a positivity effect in their cognitive performance. In the present experiment, older adults were very accurate in both conditions, which suggests that the task was not too demanding and that additional cognitive resources were still available. Despite this availability of cognitive resources, the data suggest that older adults did not use them to sustain an emotional bias. However, the results could be reconcilable with the SST when considering that specific task instructions may supplant chronically active emotion regulation goals in older adults in contrast to more open instructions (e.g., those allowing participants to view items as if watching TV; for a review, see Reed and Carstensen, 2012). In the present study, participants were instructed to respond to the emotional expression of each face, which might have hindered the processing of emotional stimuli in a motivation-based way. Previous studies that have also used specific and therefore restrictive task instructions in the domain of working memory and that have observed age-related differences in emotion-cognition interactions, have interpreted these within the SST theory (e.g., Mikels et al., 2005; Borg et al., 2011; Truong and Yang, 2014). Thus, it is important that the role of specific task instructions for age-related emotional biases is clarified in future research so that the theory’s validity can also be tested in the domain of working memory, where specific task instructions are the norm.

It should be noted that accuracy was not improved for congruent relative to non-word trials when neutral or happy faces were shown. As incongruent distractors did interfere with responses for neutral faces in younger adults and happy faces in both age groups, it appears unlikely that participants were able to ignore distractors when presented with neutral or happy faces. In contrast, it is possible that the failure to observe facilitation for neutral and happy faces was due to ceiling effects, as accuracy was very high for these faces. When responding to neutral faces, younger adults showed lower accuracy for incongruent relative to non-word trials under HE conditions, whereas older adults did not show differences in accuracy between incongruent and non-word trials. On the one hand, this seems to suggest that older adults did not rely on external cues when responding to neutral targets under conditions requiring proactive control. On the other hand, it is also possible that the task conflict created by target words and distractor faces was not high enough under conditions requiring proactive control to affect accuracy in older adults. It is not possible to disentangle these two explanations in the present paradigm. However, the result suggests that older adults were able to overcome information conflict elicited by incongruent trials under conditions requiring proactive control and highlights preserved or even improved proactive control in older relative to younger adults.

It should be noted that facilitation in RTs was found for both age groups in both conditions. This finding suggests that the priming of task-relevant processing pathways improved performance for congruent relative to non-word trials irrespective of expectancy of conflict. Although research suggests that low levels of proactive control are associated with no or even reversed facilitation (Tzelgov et al., 1992; Goldfarb and Henik, 2007; Kalanthroff et al., 2013, 2015), a review by Roelofs (2003) has shown that facilitation occurs when distractors precede target stimuli as they did in Experiment 1: participants were presented with the distractor label 100 ms before the target face appeared. According to Roelofs (2003), such a preview can prime a particular response, resulting in facilitation in congruent trials, and this effect is considered to be “automatic” with preview times under 250 ms. Thus, it appears that the implementation of a distractor-first design in Experiment 1 resulted in facilitation across both experimental conditions.

## Experiment 2

Experiment 1 showed that emotional material affected cognitive performance in an emotional Stroop paradigm. More specifically, participants responded more accurately and faster when Stroop targets were happy faces, whereas accuracy was lowest and RTs were slowest for angry faces. However, it is not clear whether these effects of emotion can be expected for other stimulus sets such as words. On the one hand, research has shown more efficient processing of emotional relative to neutral material using a wide range of stimulus sets, including faces (e.g., Juth et al., 2005; Brosch et al., 2008; Calvo and Nummenmaa, 2008), images (e.g., Fox et al., 2007; Langeslag and Van Strien, 2008; Olofsson et al., 2008) and words (e.g., Hamann and Mao, 2002; Gotoh, 2008; Kopf et al., 2013). This suggests that effects of emotion can be expected to be consistent across different stimulus sets. On the other hand, there is also evidence that orienting to affective material was more pronounced for faces than for words (Kensinger and Corkin, 2003; Vuilleumier, 2005; Kensinger and Schacter, 2006) and that enhanced processing of emotional content was automatic for faces but not for words (Rellecke et al., 2011). Such differences in the effects of emotional faces and words were usually explained by differences in extracting emotional significance from words and faces. For instance, it was suggested that words must be processed to a higher level than faces before their meaning could be assessed (Kensinger and Corkin, 2003) and that their emotional significance needs to be extracted based on semantic knowledge (Schacht and Sommer, 2009; Rellecke et al., 2011). In contrast, perceptual features are used to extract emotional significance in faces (Vuilleumier, 2005; Vuilleumier and Huang, 2009). Given these differences in the processing of emotional words and faces, it is likely that verbal stimuli affect cognitive control differently than facial stimuli.

By using verbal stimuli in the same task as in Experiment 1, the aim was to assess whether cognitive control of emotional words would be associated with comparable effects as were observed for emotional faces. Should emotional words produce similar effects as in Experiment 1, this would suggest that the valence (i.e., pleasantness) is sufficient to affect performance independently of their biological preparedness. In contrast, if differential effects of emotion were to be observed, this would suggest that stimulus features that are not shared by faces and words contribute to the effects of emotional items on cognitive control.

### Methods

#### Participants

Thirty younger (20–38 years old) and 30 older adults (63–78 years old) participated in the experiment (see Table 3 for participant characteristics). One younger and one older adult were excluded from the analysis due to RTs that were 2.5 *SD* slower than the respective group’s mean RTs. Additionally, one younger adult was excluded due to high BDI-II scores, indicating moderate levels of depression. The recruitment criteria were the same as in Experiment 1 and none of the participants had taken part in the previous experiment. As can be seen in Table 3, older adults had better verbal knowledge than younger adults as assessed with the NART (Nelson and Willison, 1991) and scored lower on the Digit Symbol Substitution Test (Wechsler, 1955), suggesting slower processing speed in older than in younger adults. Whereas these results are commonly observed in ageing research as highlighted above, it was also found that older adults reported fewer years of education than younger adults. Additionally, younger adults reported higher levels of trait anxiety than older adults as assessed by the A-Trait version of the STAI (Spielberger et al., 1983). No further differences were observed between the two age groups. Older participants had a score of 27 or above on the MMSE (Folstein et al., 1975). The ethics board of Birkbeck, University of London, approved the procedure prior to the start of the study and written informed consent was obtained from each participant.

**TABLE 3:** Participant characteristics, Experiment 2.

	**Younger adults**		**Older adults**		**Group difference**	
**Variable**	***M***	***SD***	***M***	***SD***	***t***	***p***
Age (years)	26.42	6.53	72.93	5.74		
Gender (male/female)	10/18		6/23			
Education (years)	17.57	2.73	15.71	3.46	2.26	0.028
NART Verbal IQ	106.96	6.80	119.74	13.38	–4.42	¼0.001
Digit Symbol Test	63.26	12.13	47.38	10.05	5.35	¼0.001
BDI II	6.81	4.12	6.11	4.50	0.61	0.546
STAI Trait Anxiety	38.30	7.22	32.89	9.66	2.33	0.024
MMSE			29.04	0.88		

*NART = The National Adult Reading Test, BDI II = Beck Depression Inventory II, STAI = State-Trait Anxiety Inventory, MMSE = Mini-Mental State Examination.*

#### Materials

**FIGURE 4:**
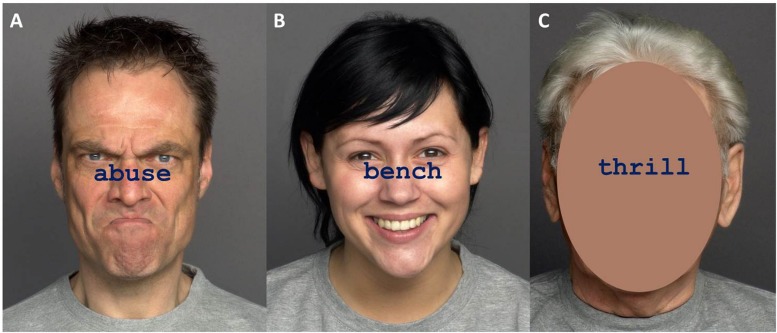
Examples of Stroop stimuli in Experiment 2. Panel **(A)** shows a negative word with a congruent face, panel **(B)** shows a neutral word with an incongruent face, and panel **(C)** shows a positive word with an obscured face (non-face condition). Pictures are taken from the FACES database (Ebner et al., 2010) and can be accessed at: https://faces.mpdl.mpg.de/imeji/. Publication and display of the shown pictures for the purpose of illustrating research methodology are permitted under the FACES Platform Release Agreement.

Stimuli consisted of a selection of 36 words from the ANEW database (Bradley and Lang, 1999), which provides normative emotional ratings for a large number of words in the English language. Words were either negative (e.g., abuse, wounds, crime), emotionally neutral (e.g., bench, board, moment) or positive (e.g., thrill, hug, love) and there were 12 words per category. The words had been rated in a preliminary evaluation study and were selected based on high agreement ratings between younger and older raters (see Supplementary Materials for evaluation details). Congruent items were created by printing the word on emotionally matching faces that were used in Experiment 1 (e.g., word “thrill” with happy face). Incongruent items were created by printing a word on non-matching emotional faces (e.g., word “bench” with angry face). “Non-face” items (equivalent to non-word items used in the previous experiments) were created by printing the word on a face picture, in which the area of the face was obscured. Combinations of words and faces are summarized in Table 4. Target words were printed in navy blue, 38-point Courier New font, and placed between the face’s eyes and mouth. The face images were colored photographs that appeared 100 ms before the word, in accordance with the procedures used in Experiment 1. Example stimuli are presented in Figure 4.

**TABLE 4:** Combinations of words and facial expressions that formed congruent, non-face and incongruent stimuli in Experiment 2.

	**Task-relevant word**		
**Distractor face**	**Negative**	**Neutral**	**Positive**
Angry	Congruent	Incongruent	Incongruent
Neutral	Incongruent	Congruent	Incongruent
Happy	Incongruent	Incongruent	Congruent
Obscured	Non-face	Non-face	Non-face

*Congruent stimuli are color-coded in green, non-face stimuli in yellow and incongruent stimuli in red.*

#### Procedure

The procedure for Experiment 2 was identical to that of Experiment 1 as were the proportions of congruent, incongruent and non-face trials in the HE and LE blocks. There were equal numbers of negative, neutral and positive words across trials of different congruencies and across the two blocks. Each trial began with the presentation of the distractor face that was happy, neutral, angry, or obscured for 100 ms, followed by the simultaneous presentation of the distractor face and the target word. Participants were instructed to indicate the emotional valence of the word (negative, neutral or positive) as accurately and quickly as possible by pressing one of three labeled buttons.

#### Design and Statistical Analysis

The recording and exclusion of data were identical as in Experiment 1. Accuracy and RTs were analyzed by 2 × 3 × 3 × 2 mixed factors ANOVA including the within-subjects factors expectancy (LE vs. HE), congruency (congruent vs. non-face vs. incongruent) and emotion (negative vs. neutral vs. positive) as well as the between-subjects factor of age (younger vs. older). Procedures to conduct *post hoc* tests and to determine significance were as described above. Due to significant differences in the two age groups’ reported years of education, verbal knowledge, processing speed and anxiety scores, all analyses were repeated with years of schooling, NART verbal IQ, Digit Symbol and STAI Trait Anxiety as centered covariates. The results with age as a factor reported here were qualitatively the same and significant in the analysis including covariates. As latencies varied considerably between younger and older adults, log-transformed RT data were used for the analysis. To aid interpretation, pre-transformed RTs are reported in the descriptives and figures.

### Results

#### Accuracy

**FIGURE 5:**
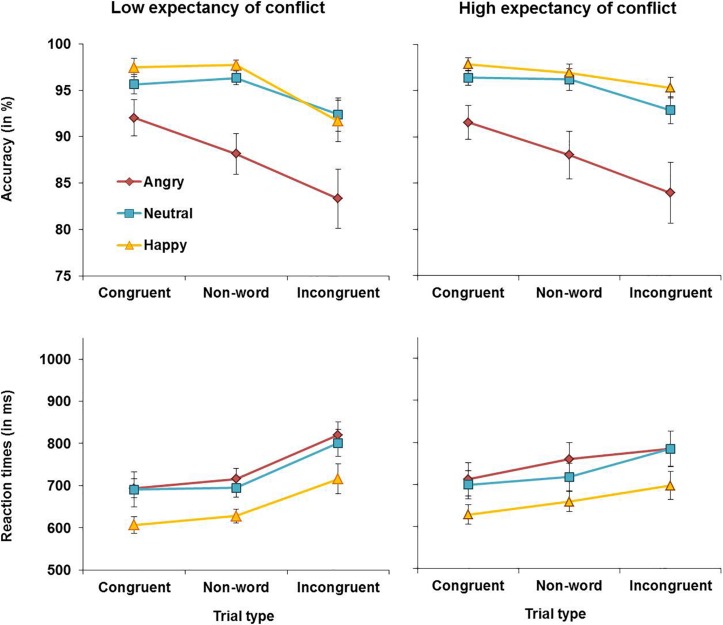
Accuracy scores in younger **(left panels)** and older adults **(right panels)** in Experiment 2.

Accuracy scores for younger and older adults are shown in Figure 5. The analysis yielded a main effect of emotion, *F*(2, 110) = 12.64, MSE = 0.081, *p* < 0.001, partial η^2^ = 0.19, as accuracy was generally higher for negative words (*M* = 97.7%, *SD* = 3.9%) than for neutral (*M* = 89.2%, *SD* = 13.2%), *t*(56) = 4.72, *p* < 0.001, or positive words (*M* = 91.7, *SD* = 7.8), *t*(56) = 6.33, *p* < 0.001. Accuracy scores for neutral and positive words were not significantly different (*p* = 0.267). There was a significant main effect of congruency, *F*(2, 110) = 7.23, MSE = 0.004, *p* = 0.002, partial η^2^ = 0.12, as accuracy was lower for incongruent trials (*M* = 91.9%, *SD* = 6.0%) than for congruent (*M* = 93.4%, *SD* = 5.2%), *t*(56) = 2.98, *p* = 0.004, or non-face trials (*M* = 93.2%, *SD* = 5.0%), *t*(56) = 2.96, *p* = 0.005. There was no difference in accuracy between non-face and congruent trials (*p* = 0.602). This main effect was qualified by a marginally significant congruency × age interaction, *F*(2, 110) = 3.13, MSE = 0.004, *p* = 0.053, partial η^2^ = 0.05, as in younger adults, accuracy was significantly higher for congruent (*M* = 93.4%, *SD* = 5.7%) relative to incongruent trials (*M* = 90.8%, *SD* = 7.1%), *t*(27) = 3.35, *p* = 0.002. In older adults, accuracy scores for congruent and incongruent trials were not significantly different (*p* = 0.451). There was also an expectancy × congruency × emotion × age interaction, *F*(4, 220) = 3.96, MSE = 0.003, *p* = 0.007, partial η^2^ = 0.07. Accuracy scores under HE and LE conditions were analyzed separately to follow up this interaction. The congruency × emotion × age interaction was significance under HE conditions, *F*(4, 220) = 3.27, MSE = 0.002, *p* = 0.019, η^2^ = 0.06, but not under LE conditions (*p* = 0.142). As a next step, younger and older adults’ data were analyzed separately and a congruency × emotion interaction was significant in younger adults, *F*(4, 108) = 2.91, MSE = 0.002, *p* = 0.039, η^2^ = 0.10, but not in older adults (*p* = 0.471). Further analyses of younger adults’ data showed that there was a main effect of congruency for neutral words, *F*(2, 54) = 6.38, MSE = 0.004, *p* = 0.003, η^2^ = 0.19, but not for negative (*p* = 0.402) or positive words (*p* = 0.372). Follow-up *t*-test indicated that under HE conditions, younger adults showed higher accuracy for congruent neutral words (*M* = 92.6%, *SD* = 14.8%) than for incongruent neutral words (*M* = 87.4%, *SD* = 15.2%), *t*(27) = 4.45, *p* < 0.001, or non-face neutral words (*M* = 91.8%, *SD* = 12.1%), *t*(27) = 2.61, *p* = 0.014. No further significant main effects or interactions were observed for accuracy.

#### Reaction Times

**FIGURE 6:**
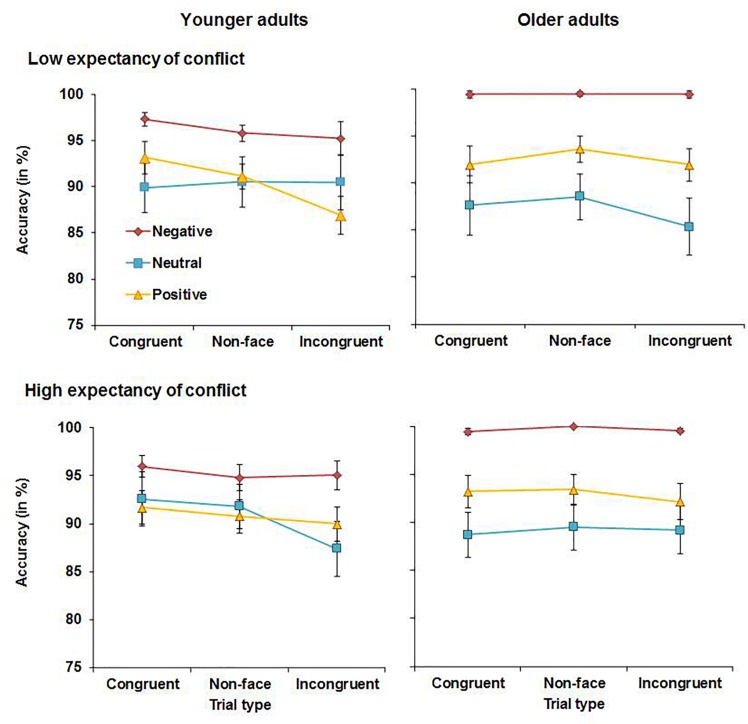
RTs for correct responses in younger **(left panels)** and older adults **(right panels)** in Experiment 2.

RTs for younger and older adults are shown in Figure 6. The analysis yielded a main effect of congruency, *F*(2, 110) = 42.70, MSE = 0.006, *p* < 0.001, partial η^2^ = 0.44, with overall slower RTs for incongruent (*M* = 802 ms, *SD* = 160 ms) than for non-face trials (*M* = 762 ms, *SD* = 142 ms), *t*(56) = 7.05, *p* < 0.001, or congruent trials (*M* = 762 ms, *SD* = 151 ms), *t*(56) = 8.42, *p* < 0.001. There was no significant difference in RTs for non-face compared to congruent trials (*p* = 0.582). This main effect was qualified by a significant expectancy × congruency interaction, *F*(2, 110) = 6.71, MSE = 0.004, *p* = 0.005, partial η^2^ = 0.11. To follow up this interaction, the analysis was repeated with the factor congruency only comprising the factor levels congruent and non-face trials, which resulted in a significant expectancy × congruency interaction, *F*(1, 56) = 7.32, MSE = 0.004, *p* = 0.009, partial η^2^ = 0.12. The analysis with the factor congruency comprising the factor levels non-face and incongruent trials also resulted in a significant expectancy × congruency interaction, which was more pronounced, *F*(1, 56) = 11.98, MSE = 0.006, *p* = 0.001, partial η^2^ = 0.18. Follow-up *t*-tests revealed that under HE conditions, RTs on congruent trials (*M* = 755 ms, *SD* = 163 ms) were slightly faster than on non-face trials (*M* = 765 ms, *SD* = 154 ms), *t*(56) = 2.24, *p* = 0.029 (marginally significant after Bonferroni correction), whereas under LE conditions, the comparison between congruent and non-face trials was not significant (*p* = 0.155). Moreover, under HE conditions, RTs were slower for incongruent (*M* = 785 ms, *SD* = 153 ms) than for non-face trials (*M* = 765 ms, *SD* = 154 ms), *t*(56) = 3.20, *p* = 0.002. Under LE conditions, the difference in RTs between incongruent (*M* = 818 ms, *SD* = 188 ms) and non-face trials (*M* = 759 ms, *SD* = 159 ms) was even more pronounced, *t*(56) = 7.16, *p* < 0.001. Additionally, there was a significant main effect of emotion, *F*(2, 110) = 13.90, MSE = 0.031, *p* < 0.001, partial η^2^ = 0.20, and follow-up analyses revealed that RTs for negative words (*M* = 746 ms, *SD* = 128 ms) were faster than for neutral words (*M* = 811 ms, *SD* = 192 ms), *t*(56) = 4.88, *p* < 0.001. RTs for positive words (*M* = 769 ms, *SD* = 155 ms) were also faster than for neutral words, *t*(56) = 3.32, *p* = 0.002, with no significant difference between RTs for positive and negative words (*p* = 0.064). Lastly, there was a main effect of age, *F*(1, 55) = 21.99, MSE = 0.437, *p* < 0.001, partial η^2^ = 0.29, as older adults were overall slower (*M* = 849 ms, *SD* = 142 ms) than younger adults (*M* = 699 ms, *SD* = 115 ms). No further significant main effects or interactions were found for RTs.

### Discussion

Experiment 2 investigated the effects of emotional target words on cognitive control in a Stroop paradigm. It was found that both younger and older adults showed reduced interference and facilitation in RTs under HE compared to LE conditions, suggesting that they engaged in proactive control when the proportion of conflict-generating trials was high. It was also found that emotion facilitated task performance in both younger and older adults, with more accurate responses to negative relative to neutral or positive words. Responses were faster for both negative and positive words relative to neutral words in younger and older adults. Age-related differences emerged for accuracy under HE conditions: When neutral words were the targets, younger adults showed lower accuracy for incongruent relative to non-face or congruent trials, whereas older adults did not show differences in accuracy between congruent, non-face and incongruent trials.

The enhancing effect of emotion on cognitive control in an emotional Stroop task was observed for both positive and negative words, as participants responded faster when presented with emotional rather than neutral words. This could be due to enhanced sensory processing of emotional material including words (Phelps and LeDoux, 2005; Vuilleumier, 2005; Phelps et al., 2006; Vuilleumier and Huang, 2009). It should be noted, however, that the enhancing effect on performance was particularly pronounced for negative words, as accuracy was higher for negative relative to neutral or positive words. This contrasts with findings from Experiment 1 showing improved performance for happy relative to neutral or angry faces. Differences in the effects of words and faces on Stroop performance will be discussed in the general discussion below.

When responding to neutral words, younger adults showed lower accuracy for incongruent relative to congruent or non-face trials under HE conditions in the present experiment. In contrast, older adults did not show differences in accuracy between congruent, non-face or incongruent trials. This mirrors the results in Experiment 1 that showed no interference effect for neutral targets in older adults and suggests that older adults were relatively more successful than younger adults in responding to neutral words without being affected by distractors under HE conditions. Thus, the data do not support the notion of reduced proactive control in aging but suggest that older adults can even outperform younger adults under conditions requiring proactive control.

## General Discussion

Two emotional Stroop experiments were conducted to assess proactive control in younger and older adults. The deployment of proactive control was manipulated by varying the expectancy of congruent and incongruent trials relative to trials without conflict (i.e., non-words in Experiment 1 and non-face items in Experiment 2). Besides addressing age-related differences in cognitive control, these experiments also investigated the effects of emotion on cognitive control using facial and verbal stimuli. These experiments revealed the following critical findings: First, older adults successfully deployed proactive control when the proportion of conflict-inducing items in a Stroop task was high. Second, emotion affected cognitive performance in a Stroop task similarly in both age groups. Third, the effects of emotion on performance were not uniform across facial and verbal stimuli. In the following, the implications of these findings will be discussed.

### No Evidence for Age-Related Impairments in Proactive Control in Emotional Stroop Tasks

The present findings extend the empirical evidence obtained in studies using the AX-CPT task (Rosvold et al., 1956) and suggest that older adults can deploy proactive control when needed. Across two experiments using an emotional Stroop paradigm, older adults showed reduced interference from incongruent relative to non-word/non-face trials when expectancy of conflict was high. Moreover, both age groups showed facilitation across both HE and LE conditions in Experiment 1 and under HE conditions in Experiment 2, with no age-related differences. These results are in accordance with prior research showing no age-related impairments in proactive control (Paxton et al., 2008, Exp. 2; Staub et al., 2014).

In contrast, these results deviate from findings of previous research with the AX-CPT task (Rosvold et al., 1956). These indicated that older adults have greater difficulties than younger adults to efficiently use the context for a target response in AX-CPT tasks and were viewed as evidence for age-related decline in proactive control (e.g., Braver et al., 2005, 2009; Haarmann et al., 2005; Paxton et al., 2008, Exp. 1). The contrasting pattern of results suggests that ageing is not associated with a general impairment in proactive control but that older adults’ ability to deploy it successfully might depend on the demand characteristics of the task at hand. The AX-CPT task can be used to assess proactive control “locally” at the level of trials, whereas the present study used a global approach to manipulate proactive control across an entire block of trials in a Stroop paradigm. Older adults were outperformed by younger adults in the former but not in the latter task. This suggests that, although they might find it difficult to adapt their performance flexibly on a trial-by-trial basis or under conditions of uncertainty (see also Mayr, 2001; Mutter et al., 2005), older adults can adapt to task conflict and deploy proactive control over a period of time (see also West and Baylis, 1998; cf. Staub et al., 2014). Moreover, participants have to maintain a two-fold set of rules and to update information in working memory in the AX-CPT task, which is not required in a Stroop task. Previous research has shown age-related impairments in maintaining multiple tasks in working memory (Verhaeghen and Cerella, 2002; Reimers and Maylor, 2005; Wasylyshyn et al., 2011) and in working memory updating (Van der Linden et al., 1994; Hartman et al., 2001; Salthouse et al., 2003; De Beni and Palladino, 2004; Chen and Li, 2007; Schmiedek et al., 2009). Thus, it is possible that older adults showed no impairments in proactive control in the Stroop but in the AX-CPT task as the latter additionally involves processes that are known to undergo age-related changes.

### Stimulus-Specific Effects of Emotion on Cognitive Control

The experiments assessed the effects of emotion on cognitive control in younger and older adults. In Experiment 1, emotional faces were used as targets and happy faces were found to improve both accuracy and RTs in younger and older adults. This finding is in line with previous literature showing improved performance for happy faces relative to other expressions in WM tasks with facial stimuli (Levens and Gotlib, 2010, 2012; Cromheeke and Mueller, 2015). The effects of emotion were largely consistent across conditions, suggesting that emotion affected more general processes rather than cognitive control *per se*. More specifically, it is likely that improved performance for happy faces was driven by a recognition advantage of happy faces (Juth et al., 2005; Becker et al., 2011; Becker and Srinivasan, 2014) and their overall rewarding effect (O’Doherty et al., 2003; Tsukiura and Cabeza, 2008) as discussed above. Importantly, the facilitating effects of emotion did not differ in the two age groups and there was no evidence for an increased positivity effect in ageing. This finding is not fully compatible with SST (Carstensen, 1993), according to which older adults focus on positive information in order to improve their wellbeing. However, it has been argued that the positivity effect emerges under instructions encouraging participants to process material freely (for a review, see Reed and Carstensen, 2012). In the present experiments, participants received specific instructions how to respond to stimuli, giving less room for older adults to process material the way they wanted. Thus, it is possible that their chronically active bias to focus on positive material was overridden by specific task requirements in Experiment 1.

In contrast to the facilitating effects of happy faces and impairing effects of angry faces, a somewhat reversed pattern of results was found for emotional words in Experiment 2. Both younger and older adults responded more accurately to negative relative to neutral and positive words, whereas RTs were faster for both negative and positive words relative to neutral words. Together, the results from Experiment 1 and 2 add to growing evidence that the effects of emotion on cognitive performance are not consistent across different stimulus sets. Previous studies focused on the processing of emotional stimuli (Kensinger and Schacter, 2006; Rellecke et al., 2011) as well as attention (Vuilleumier, 2005; Vuilleumier and Huang, 2009) and reported that orienting to emotional material was more pronounced for faces than for words (Vuilleumier, 2005; Kensinger and Schacter, 2006; Rellecke et al., 2011). Such differences were usually explained with reference to the biological preparedness of emotional faces in contrast to words. More specifically, it was suggested that differences arise as emotional significance of faces can be extracted from perceptual features (Vuilleumier, 2005; Vuilleumier and Huang, 2009), whereas emotional significance needs to be extracted based on semantic knowledge from words (Kensinger and Corkin, 2003; Schacht and Sommer, 2009; Rellecke et al., 2011).

Consistent with previous research that found a stronger effect for faces than for words on cognitive performance (Kensinger and Corkin, 2003) the present research showed that the effects of emotion were greater for emotional faces in Experiment 1 (Accuracy: η^2^ = 0.34; Reaction times: η^2^ = 0.48) than for emotional words in Experiment 2 (Accuracy: η^2^ = 0.19; Reaction times: η^2^ = 0.20). However, the effects differed not only in size but also in their overall qualitative pattern. It is possible that extracting emotional significance by using semantic knowledge modified the effects of emotion on cognition not in a quantitative but a qualitative way. To gain a better understanding of why differences in effects between facial and verbal stimuli were observed, emotional pictures could be used in future studies. These allow the extraction of emotional significance through perceptual features but can convey the same meaning as words (e.g., picture of bomb rather than word “bomb”). Similar findings between pictures and words would indicate that faces are special in their effect on cognitive performance, which could be due to their evolutionary importance. In contrast, similar effects between pictures and faces would suggest that the extraction of emotional significance through perceptual features or semantic knowledge is relevant for the effect of emotion on cognitive performance.

Facilitation in RTs (i.e., faster RTs for congruent relative to non-word trials) was found for both age groups across both conditions in Experiment 1, which is consistent with Roelofs (2003) suggestion that preceding distractors can prime a response. However, despite the same distractor-first paradigm in Experiment 2, facilitation was eliminated under LE relative to HE conditions. A reason for varying findings for facilitation in the two experiments could lie in differences in distractor priming between the different stimulus sets. In Experiment 1, irrelevant *labels* were presented 100 ms before the target face, whereas in Experiment 2, irrelevant *faces* were presented 100 ms before the target word. It is possible that priming was more effective for label than for face distractors for several reasons. For instance, the verbal modality of label distractors was congruent with the modality of responses in Experiment 1, as participants were required to respond to faces by using labels (“happy,” “neutral” or “angry”). In contrast, there was no modality congruency between face distractors and target responses using labels (“positive,” “neutral” or “negative”) in Experiment 2. It is also possible that priming of words was particularly efficient in Experiment 1, as participants’ attention was already directed to the word by the previously presented fixation cross. In contrast, the area of the face that the participants’ attention was directed to by the fixation cross in Experiment 2 was unlikely the most diagnostic one as it was in the face’s center rather than in the eye or mouth region. Thus, participants would have needed to saccade to the eye or mouth region to assess the expression in a short period of time. Taken together, it appears that despite using the same distractor-first design in both experiments, distractor-first priming was more efficient in Experiment 1 with target faces and distractor labels than in Experiment 2 with target words and distractor faces.

## Conclusion

The present study contributes to research on proactive control in ageing and its effectiveness in the presence of emotional material. No age-related differences in proactive control were found in an emotional Stroop paradigm, which contrasts with results from AX-CPT studies that found age-related impairments in proactive control. Moreover, it was found that task-relevant emotion affected performance similarly in younger and older adults and that the effects of emotion on performance were qualitatively different for emotional faces and emotional words. Overall, these results highlight that the effects of emotion and age on proactive control depend on the task at hand and the chosen stimulus set.

## Data Availability

The datasets generated for this study are available on request to the corresponding author.

## Ethics Statement

All subjects gave written informed consent in accordance with the Declaration of Helsinki. The protocol was approved by the ethics board of Birkbeck, University of London.

## Author Contributions

NB contributed to the conception and design of the work, data collection, data analysis, and interpretation, drafting of the manuscript. AR and ED contributed to the conception and design of the work, the interpretation of the data, and critical revision of the manuscript.

## Conflict of Interest Statement

The authors declare that the research was conducted in the absence of any commercial or financial relationships that could be construed as a potential conflict of interest.
