# Supplementary material for: Preserved Proactive Control in Ageing: A Stroop Study With Emotional Faces vs. Words
Source: Front Psychol. 2019 Sep 3;10:1906. doi: 10.3389/fpsyg.2019.01906 (PMC6733973; doi:10.3389/fpsyg.2019.01906)
Supplement: Supplementary file 1 [file Data_Sheet_1.docx]

## Supplemental material

### Evaluation of the stimulus set used in Experiment 2

Forty younger (19 – 37 years old; *M* = 26.80, *SD* = 6.12) and 40 older adults (62 – 78 years old; *M* = 71.64, *SD* = 5.53) rated a total of 300 preselected words from the ANEW database (Bradley & Lang, 1999). The stimuli were divided in two sub-sets of 150 words, respectively, and each sub-set was rated by 20 younger and 20 older adults. In each sub-set, 50 words were negative, 50 were neutral and the remaining 50 were positive. Assignment to the emotional categories was based on the evaluation ratings by students that were provided in the ANEW database. The words were rated on a 9-point Likert scale for valence (1 = *very unpleasant*; 9 = *very pleasant*) and arousal (1 = *not arousing*; 9 = *very arousing*).

Mean arousal and mean valence ratings for the 150 words of each set were submitted to a 3 × 2 mixed factors ANOVA including the within-subjects factor of stimulus emotion (positive, neutral, negative) and the between-subjects factor of age of rater (younger, older), respectively. Post-hoc t-tests with a Bonferroni adjustment to the alpha level were performed to follow up significant effects and interactions.

#### **Set 1**

The analysis of arousal ratings yielded a significant effect of stimulus emotion, *F*(2, 76) = 25.64, *MSE* = 3.46, *p* < .001, partial *η^2^* = .40, with negative words receiving higher arousal ratings (*M* = 6.73, *SD* = 2.18) than neutral words (*M* = 4.64, *SD* = 1.76), *t*(39) = 5.08, p < .001. Positive words also received higher arousal ratings (*M* = 6.82, *SD* = 1.71) than neutral words, *t*(39) = 11.06, *p* < .001, whereas arousal ratings for negative and positive words were not significantly different (*p* = .816). No further main effects or interactions were observed for arousal ratings.

The analysis of valence ratings yielded a significant effect of stimulus emotion, *F*(2, 76) = 613.16, *MSE* = 0.71, *p* < .001, partial *η^2^* = .94, as negative words received lower valence ratings (*M* = 2.00, *SD* = 1.03) than neutral words (*M* = 5.48, *SD* = 0.67), *t*(39) = 18.24, *p* < .001, or positive words (*M* = 7.94, *SD* =0.54), *t*(39) = 31.99, *p* < .001. Positive words also received higher valence ratings than neutral words, *t*(39) = 20.03, *p* < .001. No further main effects or interactions were observed for valence ratings.

#### **Set 2**

The analysis of arousal ratings yielded a significant main effect of stimulus emotion, *F*(2, 76) = 119.71, *MSE* = 1.36, *p* < .001, partial *η^2^* = .76, as negative words received higher arousal ratings (*M* = 6.23, *SD* = 1.43) than neutral words (*M* = 3.07, *SD* = 1.51), *t*(39) = 11.73, p < .001. Positive words also received higher arousal ratings (*M* = 5.97, *SD* = 1.44) than neutral words, *t*(39) = 12.51, *p* < .001, whereas arousal ratings for negative and positive words were not significantly different (*p* = .092). No further main effects or interactions were observed for arousal ratings.

The analysis of valence ratings yielded a significant effect of stimulus emotion, *F*(2, 76) = 480.72, *MSE* = 0.82, *p* < .001, partial *η^2^* = .93, as negative words received lower valence ratings (*M* = 2.38, *SD* = 0.76) that neutral words (*M* = 5.18, *SD* = 0.25), *t*(39) = 23.58, *p* < .001, or positive words (*M* = 7.25, *SD* =0.73), *t*(39) = 23.31, *p* < .001. Positive words also received higher valence ratings than neutral words, *t*(39) = 16.92, *p* < .001. No further main effects or interactions were observed for valence ratings.

#### **Final selection of words**

As these analyses showed, no interactions between the factors stimulus emotion and age of rater were found, suggesting that ratings for negative, neutral and positive words did not significantly differ between younger and older adults. To select the final stimulus set, valence and arousal ratings were ranked according to difference scores of younger and older adults’ ratings. Thirty-six items with the lowest difference between ratings of younger and older adults were then selected.

The final selection of 36 words comprised 12 words per emotional category. For this selection, valence ratings for negative words were lower (*M* = 1.96, *SD* = .96) than valence ratings for neutral words (*M* = 5.47, *SD* = .74), whereas valence ratings for neutral words were lower than valence rating for positive words (*M* = 7.90, *SD* = .82). Moreover, arousal ratings were higher for negative words (*M* = 6.66, *SD* = 1.85) than for neutral words (*M* = 3.73, *SD* = 2.02), but were similarly high as those for positive words (*M* = 6.77, *SD* = 1.54). No inferential tests were conducted due to the fact that different groups of participants have evaluated different sub-sets of words. Neither an independent-samples nor a paired-samples *t*-test were appropriate to analyse mean differences for valence and arousal ratings and thus, only descriptive statistics are provided.

**References**

Bradley, M. M., & Lang, P. J. (1999). Affective norms for English words (ANEW): Instruction manual and affective ratings. *Technical Report C-1*: The Center for Research in Psychophysiology, University of Florida.
